# Investigating the neuroprotective potential of rAAV2‐PCBP1‐EGFP gene therapy against a 6‐OHDA‐induced model of Parkinson's disease

**DOI:** 10.1002/brb3.3376

**Published:** 2024-01-29

**Authors:** Ling‐Yun Ma, Lanying Wang, Jiantao Liang, Lirong Huo

**Affiliations:** ^1^ Central Laboratory Department of Neurology Fuxing Hospital, Capital Medical University Beijing China; ^2^ Department of Neurobiology Capital Medical University Beijing China; ^3^ Department of Microbiology and Immunology Medical College of Shanxi Medical University Taiyuan China; ^4^ Department of Neurosurgery Xuanwu Hospital, Capital Medical University Beijing China

**Keywords:** 6‐OHDA, gene therapy, Parkinson's disease, poly(rC)‐binding protein 1, recombinant adeno‐associated virus

## Abstract

**Objectives:**

Previous studies have suggested a potential link between poly(rC)‐binding protein 1 (PCBP1) and neurodegenerative diseases, including Parkinson's disease (PD). However, the precise role of PCBP1 in the pathogenesis of PD remains unclear. Therefore, the main objective of this study was to investigate the neuroprotective effects of PCBP1 in a PD model.

**Methods:**

To evaluate the neuroprotective potential of PCBP1, we conducted cell count assays and observed the expression of heat shock protein 70 (HSP70) in SH‐SY5Y cells exposed to 6‐OHDA‐induced neurotoxicity. Additionally, we utilized recombinant adeno‐associated virus (rAAV2) vectors encoding PCBP1 or EGFP, which were injected into the rat striatum. After 2 weeks of vector or saline injection, 6‐OHDA was administered to the rat striatum. Behavioral assessments using the open field test (OFT) were performed weekly for 7 weeks. At the seventh week after 6‐OHDA injection, immunohistochemistry and protein expression analyses were conducted in the three groups.

**Results:**

The results indicated that PCBP1 treatment significantly reduced the proliferation of 6‐OHDA‐induced SH‐SY5Y cells. Additionally, in surviving cells, overexpression of PCBP1 enhanced the expression of HSP70. Similarly, rAAV2 vectors effectively delivered PCBP1 into the brain, resulting in sustained expression of rAAV2‐PCBP1‐EGFP. In the OFT, PCBP1 exhibited significant improvements in behavioral abnormalities and reduced anxiety in the PD model rats (*p* < .01). Moreover, PCBP1 effectively prevented the decrease of tyrosine hydroxylase and HSP70 expression in the lesioned side induced by 6‐OHDA (*p* < .01). Consistent with expectations, PCBP1 efficiently protected against cell death caused by 6‐OHDA (*p* < .01).

**Conclusions:**

In conclusion, our findings provide compelling evidence for the beneficial effects of PCBP1 in the PD model, suggesting that PCBP1 could be a potential therapeutic target for PD.

## Graphical abstract

The graphic abstract outlines the chronological sequence of experimental procedures for surgery and testing across the three animal groups. In the neuroprotection study, lesioning of the midbrain dopaminergic (DAergic) system occurred two weeks after viral vector injections using 6‐OHDA. Following this, Open Field Testing (OFT) observations were conducted weekly for seven consecutive weeks, with continuous recording. Subsequently, euthanasia was administered, and relevant immunohistochemical (IHC) and Western blot (WB) parameters were assessed.

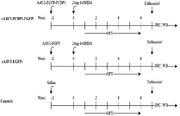

## 1 INTRODUCTION

Parkinson's disease (PD), a progressive neurodegenerative condition primarily affecting the midbrain, is the second most leading cause of neurodegenerative disorder in the elderly population, following Alzheimer's disease (AD), whereas the etiological factors largely remain unknown (Tysnes & Storstein, 2017). To date, genetic mutation is considered the most common etiology of PD pathogenesis. However, pathogenic variations in 20 monogenic loci can only account for 10%–20% of PD onsets (Blauwendraat et al., 2020). In addition, the transcriptional and posttranscriptional regulations also play important roles in PD pathophysiology (Blackinton et al., 2009).

As is widely recognized, RNA‐binding proteins (RBPs) interact with their target RNAs through highly conserved RNA‐binding domains to modulate the functions of these RNAs. Among them, poly(rC)‐binding protein 1 (PCBP1), a prominent member of the PCBP family of proteins, is extensively expressed in various human tissues. PCBP1 plays a significant role in regulating transcription and RNA metabolism at multiple levels (Hanson et al., 2012). Additionally, previous research has highlighted the regulatory role of PCBP1 in the circadian clock (Jo et al., 2020) and its involvement in maintaining iron homeostasis (Klim et al., 2021). Moreover, abnormal nuclear distribution of PCBP1 has been implicated in the pathogenesis of Huntington's disease (HD), suggesting that RBPs, including PCBP1, may play a role in the pathophysiological processes underlying neurodegenerative conditions (Geuens et al., 2017). Given the shared pathophysiology between PD and HD, PCBP1 may also be closely associated with PD, although the exact mechanism remains unclear (Wu et al., 2021). However, to date, no animal experiments have been conducted to investigate the specific mechanism of PCBP1 in regulating the process of PD models.

Studies show that recombinant adeno‐associated virus (rAAV2) can be expressed in various brain regions, including the hippocampus, substantia nigra, cortex, striatum, hypothalamus, globus pallidus (GP), subthalamic nucleus, facial nerve, and spinal cord (Huang & Kamihira, 2013). Differences in morphology, structure, and cell tropism result in varying cell types and infection efficiency among different serotypes. Although AAV1, AAV2, AAV4, and AAV5 can transduce cells of the central nervous system, they exhibit differences in infection pathways and target cell types. Among these, rAAV2 stands out as the most extensively studied serotype with a broad range of cell tropism, making it the preferred carrier for gene therapy of PD (Blesa et al., 2012). Currently, it is considered the top choice for PD gene therapy, and it has been widely investigated and utilized in clinical gene therapy trials.

Thus, HeLa S3 PCBP1‐KO cells exhibit reduced apoptosis upon exposure to hydrogen peroxide (H_2_O_2_), suggesting that PCBP1 favors apoptosis under oxidative stress conditions (Ishii et al., 2020), and oxidative stress is involved in the neurotoxic damage inflicted by 6‐OHDA (Hernandez‐Baltazar et al., 2017; Simola et al., 2007; Varešlija et al., 2020). On the other hand, the specific deletion of PCBP1 in the livers of mice was accompanied of mitochondrial dysfunction in hepatocytes, characterized by reduced levels of respiratory complexes II and IV, and ATP production (Jadhav et al., 2021). Indeed, in the preliminary stages of our research, we conducted cell experiments to explore the initial effects. As PCBP1 is an iron chaperone, relevant study proposed that the lack of PCBP1 gave rise to higher levels of chemically reactive iron, which triggered an increase of reactive oxygen species that caused the mitochondrial damage. This study relevant with our study because iron accumulation in the substantia nigra has been implied in the pathophysiology of PD (Riederer et al., 2023; Youdim et al., 1993). Another study showing the role of PCBP1 in the regulation of genes associated with inflammation and ubiquitination (Yusufujiang et al., 2022), which have been implied in the pathogenesis of neurodegenerative disorders.

HSP constitutes a family of molecular chaperones that facilitate the maintenance of native conformations in non‐native proteins by binding to them, thus preventing their aggregation or misfolding (Hu et al., 2021; Paul & Mahanta, 2014). In a study conducted by Jun and colleagues, they employed adenoviral vectors carrying HSPA1A (encoding heat shock protein 70 [HSP70]) in gene therapy, which was applied to PD model induced by 1‐methyl‐4‐phenylpyridimium (MPP+). The results revealed that the expression of HSP70 significantly protected neurons damaged by MPP+ (Jung et al., 2022). Moreover, our previous research has demonstrated that PCBP1 can modulate the expression of HSPA6 (NM_002155) and HSPA1A (NM_005345) within the HSP70 family, with the overexpression of PCBP1 leading to a marked enhancement in HSP70 expression levels (Huo & Zhong, 2008).

Consequently, we hypothesized that PCBP1 may ameliorate PD symptoms in the 6‐OHDA‐induced PD model. To investigate this, we first explored the potential pre‐protective effect of PCBP1 in the PD cell model. Furthermore, we have also observed that the overexpression of PCBP1 results in an augmentation of HSP70 expression. Subsequently, we evaluated the impact of PCBP1 on motor function and its ability to protect tyrosine hydroxylase (TH) neurons in PD rats. In addition, we analyzed the protein expression following gene transfer using Western blot (WB) and assessed TH and HSP70 expression in the substantia nigra pars compacta (SNc) region to compare the neuroprotective effect of rAAV2‐PCBP1‐EGFP gene therapy with that of rAAV2‐EGFP and saline (control). Our main objective was to examine whether rAAV2‐PCBP1‐EGFP treatment exhibits a distinct neuroprotective effect on TH in the SNc region of the PD rat model. Throughout our study, we observed behavioral improvements in a motor paradigm and the exacerbation of psychiatric‐like phenotypes. Therefore, our primary focus was on behavioral symptoms in PD, and the current study aimed to explore the potential neuroprotective role of PCBP1 against locomotor behavior and neurons in 6‐OHDA‐induced PD models in both cells and rats.

## 2 METHODS

### 2.1 In vitro assay

#### 2.1.1 Cell culture and drug treatments

SH‐SY5Y human neuroblastoma cells (ATCC) were cultured in DMEM/F12 medium supplemented with 10% heat‐inactivated fetal bovine serum and 100 μg/mL streptomycin. The cells were maintained at 37°C with 5% CO_2_ and the culture medium was refreshed every other day. Passaging of the cells was performed every 2–3 days, and cells at passage 3 were used for subsequent experiments.

To induce neurotoxicity, SH‐SY5Y cells were exposed to 25 μM of 6‐OHDA (Sigma) for 24 h. Prior to 6‐OHDA exposure, SH‐SY5Y cells were transfected with plasmids carrying PCBP1 and empty vectors using Lipofectamine 3000 (Invitrogen) 24 h before the 6‐OHDA treatment. After 36 h, the cell growth of SH‐SY5Y cells was assessed as described below.

#### 2.1.2 Proliferation assays

Cell growth was also detected by the cell counted assay (Navarra et al., 2015) by plating and treating the cells as already described.

#### 2.1.3 Real‐time polymerase chain reaction (PCR) analysis

Total RNA from the cells was extracted using the TRIzol RNA isolation reagent (Invitrogen) following the manufacturer's instructions. The RNA yield was quantified using NanoDrop 1000, and the RNA purity was assessed based on the A260/A280 ratio. Glyceraldehyde 3‐phosphate dehydrogenase (GAPDH) was used as the endogenous control.

PCR amplification was conducted with some modifications to previously reported studies. The reactions were performed using 10 μL of a real‐time mix, which included 5 μL of SYBR Green master mix (Applied Biosystems), 1 μL of cDNA, 2 μL of nuclease‐free water, 0.5 μL each of forward and reverse primers, and the following primer pairs: PCBP1 for ttctagtcgacatggatgccggtgtgactgaaagt, PCBP1 rev ttctaagatctctagctgcaccccatgcc; GAPDH for tcaacagcaactcccactctt, GAPDH rev ccagggtttcttactccttgg, and 1 μL of RNase inhibitor. The samples were initially incubated at 50°C for 2 min, followed by denaturation at 95°C for 10 min. This was followed by 40 cycles of amplification at 95°C for 15 s, 60°C for 1 min, and 72°C for 40 s (Peinnequin et al., 2004). The abundance or decline of mRNA was normalized to the geometric average of endogenous control GAPDH for Δ*Ct*. The fold change was calculated using 2^−ΔΔ^ *^Ct^* method and reported as fold change unit. For cDNA preparation, reverse transcription was carried out.

#### 2.1.4 Immunofluorescence

Immunofluorescence (IF) staining was performed following standard procedures. Cells were incubated with HSP70 polyclonal antibody (1:100 dilution, Cell Signaling Technology). After incubation with primary antibodies, the sections were washed with phosphate‐buffered saline (PBS) and incubated with the appropriate fluorescent secondary antibodies. Sections were mounted using 4′,6‐diamidino‐2‐phenylindole (Molecular Probes) and imaged by fluorescent microscopy. IF images were obtained with confocal microscopy (Leica).

### 2.2 Animal

Experiments were performed using adult male SD rats (200–230 g), purchased from Beijing Weitong Lihua Experimental Animal Technology Co., Ltd. Animals were maintained at 25°C with free access to water and food, under a 12:12 h light/dark cycle, with lights on at 7:00 am. All experiments were carried out during the light phase of the day. All efforts were made to minimize animal suffering and to reduce the number of animals used. All procedures concerning animals were carried out in accordance with animal experimentation and care and were approved by the committee of institutional animal care and use. Experimentation was carried out at the capital medical University.

#### 2.2.1 rAAV2 vector injection

After adaption for 7 days, rats were anaesthetized with a 50 mg/kg sodium pentobarbital intraperitoneal (IP) injection and placed in a stereotaxic device. After anesthesia, the rats were fixed in a stereotaxic frame by their nose and ears.

For find the most suitable titer, rats were randomly divided into 4 groups (*n* = 5–6/group) receiving three different titers of rAAV2‐EGFP (2.5 × 10^8^, 5.0 × 10^8^, 7.5 × 10^8^, 1.0 × 10^9^ vg/striatum) (Catalog 240071, Agilent Technologies). Rats were anesthetized and the recombinant rAAV2 viral vectors were injected into the rat striatum in a stereotaxic operation. Rats were anesthetized as previously described, and microinjection was performed using a 10 μL syringe (Hamilton) with the following coordinates relative to bregma, based on the stereotaxic atlas of Paxinos and Watson: anteroposterior (AP), −1.0 mm; mediolateral (ML), ± 3.5 mm; dorsoventral (DV), −4.5 mm. A total of 12 μg/2 μL of 6‐OHDA was infused on each side at a flow rate of 0.5 μL/min. The needle was kept in place for an additional 4 min before being withdrawn at a rate of 1 mm/min.

In order to evaluate the expression of the virus vector in a titer‐dependent and time‐dependent manner, we conducted a series of experiments in rats. Different titers of the rAAV2 vector were injected into the rats, and they were sacrificed at various time points, namely, 1, 2, 3, 4, 5, 6, and 7 weeks after injection. Prior to sacrifice, the rats were anesthetized with IP anesthesia. The brains of the rats were then fixed by intracardial perfusion with 4% paraformaldehyde for 10 min, followed by an additional 4 h of postfixation while floating in paraformaldehyde. After being stored in sucrose, the brains were frozen and sliced into 40‐μm‐thick sections in sets of six using a sliding microtome. The expression of AAV2‐EGFP in the rat brain sections was observed using confocal microscopy. As a preliminary investigation, we observed stable expression of AAV2‐EGFP in the neurons of various brain regions, including the striatum, hippocampus, substantia nigra, and cortex (Figure S1).

For behavioral experiments and subsequent studies after lesioning, the rats were randomly divided into three treatment groups: 25 rats in the rAAV2‐PCBP1‐EGFP group, 25 rats in the rAAV2‐EGFP group, and 10 rats in the control group. In the rAAV2‐PCBP1‐EGFP group, rats were injected with rAAV2‐PCBP1‐EGFP (5.0 × 108 viral genomes per striatum), and in the rAAV2‐EGFP group, rats were injected with rAAV2‐EGFP (5.0 × 108 viral genomes per striatum) into the left striatum. The right striatum was left intact. Rats in the control group received a subcutaneous injection of 2 μL saline on the left side. Postoperative pain was managed with subcutaneous tramadol at a dosage of 1 mg/kg, and rats were kept individually housed overnight.

#### 2.2.2 Lesions

For all rats in the neuroprotection study, lesioning of the midbrain DAergic system was done 2 weeks after viral vector injections using 6‐OHDA (Figure 2a). Thirty minutes before the 6‐OHDA injections, rats received desipramine 15 mg/kg intraperitoneally (i.p.) (desipramine hydrochloride, Sigma) to protect noradrenergic nerve terminals from the toxin. 6‐OHDA was injected under anesthesia using stereotaxis as described above. Rat received two injections, each 10 μg of 6‐OHDA (2.5 μg/μL, 1 μL/min), into the left and right striatum according to following coordinates: AP, −1.0 mm; ML, ±3.5 mm; DV, −4.5 mm.

#### 2.2.3 Open field test (Cui et al., 2015)

To acclimate to the testing environment, the rats were transferred to the behavioral science laboratory 30 min before the experiments. Each rat was then placed in the center of the TruScan test box, and the door was closed. The rats’ spontaneous movements, distance traveled, and trajectory were recorded using the infrared detector of the TruScan Photobeam Activity System (measuring 6 × 16 × 16 in.; Coulbourn Instruments) under quiet experimental conditions. To avoid any potential influence from the scent of previously tested rats, the test box was cleaned with 10% ethanol before each trial.

Several parameters were measured during the open field test (OFT), including:

Total distance: The distance covered by the rat within the entire field.

Center distance: The number of times the rat entered the central area of the open field.

Vertical plane (VP) time: The total time the rat spent in the VP, which refers to rearing behavior.

VP entries: The total number of times the rat entered the VP.

All data were automatically calculated and recorded for each rat using the same video tracking system.

#### 2.2.4 immunohistochemistry

Following the survival period, rats were anaesthetized with an IP injection of sodium pentobarbital (60 mg/mL). Perfused intracardially initially with 0.9% saline (chilled) followed by 4% paraformaldehyde (chilled) prepared in 0.1 M PBS, pH 7.4, and then decapitated. Brains were extracted and postfixed in 10% paraformaldehyde overnight and later cryoprotected in 30% sucrose solution. Immunohistochemistry and image analysis were performed to measure and analyze the mean optical density (OD) for PCBP1. The unbiased stereological assessment of the total count of TH‐reactive cells in the SNc was conducted employing the optical fractionator method (Gorbatyuk et al., 2008). The sections were rinsed thrice for 10 min each with 0.01 M PBS (pH 7.4) and then blocked with 10% NGS in PBS 0.3% Triton‐X 100 and 1% BSA in PBS with Triton X‐100 (PBST), as blocking reagent for about 1 h. The sections were further incubated with primary antibody (polyclonal antimouse antibody against TH in 1:1000 dilution, Abcam Company; polyclonal antibodies goat anti‐hnRNP E1 in 1:1000 dilution, Santa‐Cruz Biotechnology) for 16 h at 4°C. On the next day, the sections were washed three times with PBS for 15 min and were exposed to goat anti‐mouse immunoglobulin G (IgG) secondary antibody (1:200) and anti‐rabbit IgG secondary antibody (1:200) for 120 min at room temperature. After three washes with PBS (5 min each), all sections were treated with 3,3‐diaminobenzidine and H_2_O_2_ at room temperature for 15 min. The sections washed with tap water and were counterstained with Hematoxylin, dehydrated in increasing graded alcohols, cleared in xylene, and mounted with cover slide. Bright‐field microscopy (Olympus) was performed to visualize the numbers of positive cells at 400× magnifications after diaminobenzidine staining.

We used ImageJ software (NIH) by measuring the OD of PCBP1 in the striatum and counting the number of TH‐positive SNc. The OD of PCBP1 as the sum of the OD values of PCBP1 on that side divided by the area of the effective target distribution region. The mean values of the OD were calculated from three consecutive slices for each animal's analysis. SNc were counted bilaterally in six sections (40‐μm sections, every sixth section) from each brain, ranging from approximately 4.5 to 6.0 mm posterior to bregma (Paxinos & Watson, 1997). Results are given as ratio of cells in the lesioned rat SNc as compared with the intact SNc.

### 2.3 Western blotting analyses

Tissues were homogenized in ice‐cold whole cell RIPA lysis buffer (Beyotime), and the protein concentrations were quantified using the BCA Protein Assay Kit (Beyotime). Samples were separated by 8%–12% sodium dodecyl sulfate–polyacrylamide gel electrophoresis and transferred to polyvinylidene fluoride membrane (Merk KGaA) at 4°C. After incubation with primary anti‐TH antibody (1:10,000, Sigma‐Aldrich), HSP70 polyclonal antibody (1:100 dilution, Cell Signaling Technology), and β‐actin (1:10,000, BIODESIGN). Membranes were incubated with peroxidase‐conjugated goat anti‐rabbit or anti‐mouse IgG secondary antibody (1:1000, Thermo) for 2 h at room temperature, and then protein bands were visualized with an enhanced ECL reagent. ImageJ was used to analyze the gray scale ratio of WB.

### 2.4 Statistical analysis

Statistical analyses of the behavioral and histological data were performed using GraphPad Prism software (ver. 5; GraphPad Software, Inc.). For normally distributed data, differences between treatment groups were determined using one‐way analysis of variance (ANOVA) followed by the Tukey honestly significant difference (HSD) post hoc test. Two‐way ANOVA with the Holm‐Sidak post hoc test (*α* =  .05) was used to analyze the OFT. All results are presented as mean ± standard error mean. *p*‐Values less than .05 were considered statistically significant.

## 3 RESULTS

**FIGURE 1:**
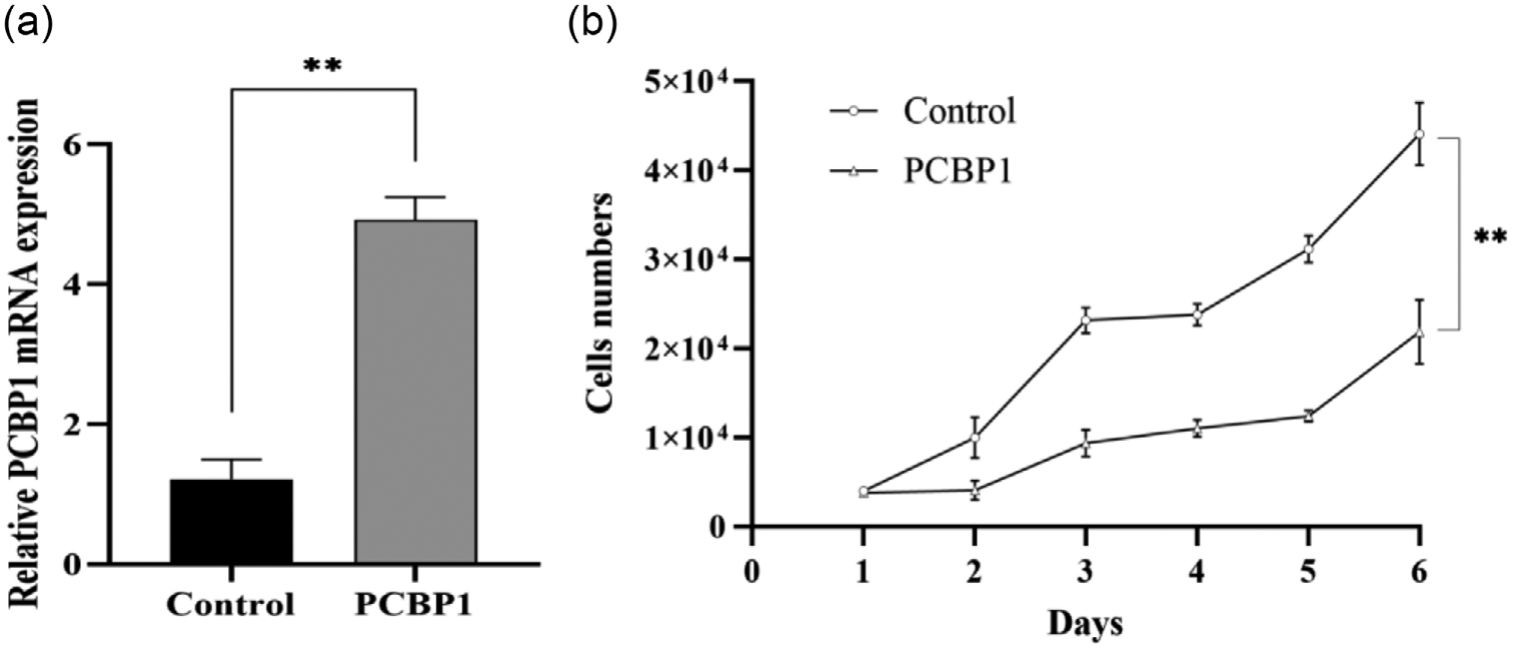
Effect of poly(rC)‐binding protein 1 (PCBP1) on 6‐OHDA‐induced apoptosis in SH‐SY5Y cells. (a) Relative mRNA expression is expressed as the ratio of PCBP1 mRNA to the amount of glyceraldehyde 3‐phosphate dehydrogenase (GAPDH) mRNA; (b) effects of 6‐OHDA, and 6‐OHDA in the presence of PCBP1 on SH‐SY5Y cells. Values shown are mean ± standard error mean (SEM). ^**^ *p* < .01 versus control group.

In Figure 1a, we demonstrated successful transfection of PCBP1 into the cells with significant expression (*p* < .01). Furthermore, the cells were transiently transfected with the PCBP1‐pcDNA3.1 recombinant plasmid and the empty PCDNA3.1 vector as the control group. On the second day, 25 μM of 6‐OHDA was added to the cells. After 24 h of 6‐OHDA treatment, cell counting was initiated. Remarkably, cells transfected with the PCBP1 recombinant plasmid showed a significantly enhanced growth rate compared to the control group, and this difference peaked on the sixth day (*p* < .01).

**FIGURE 2:**
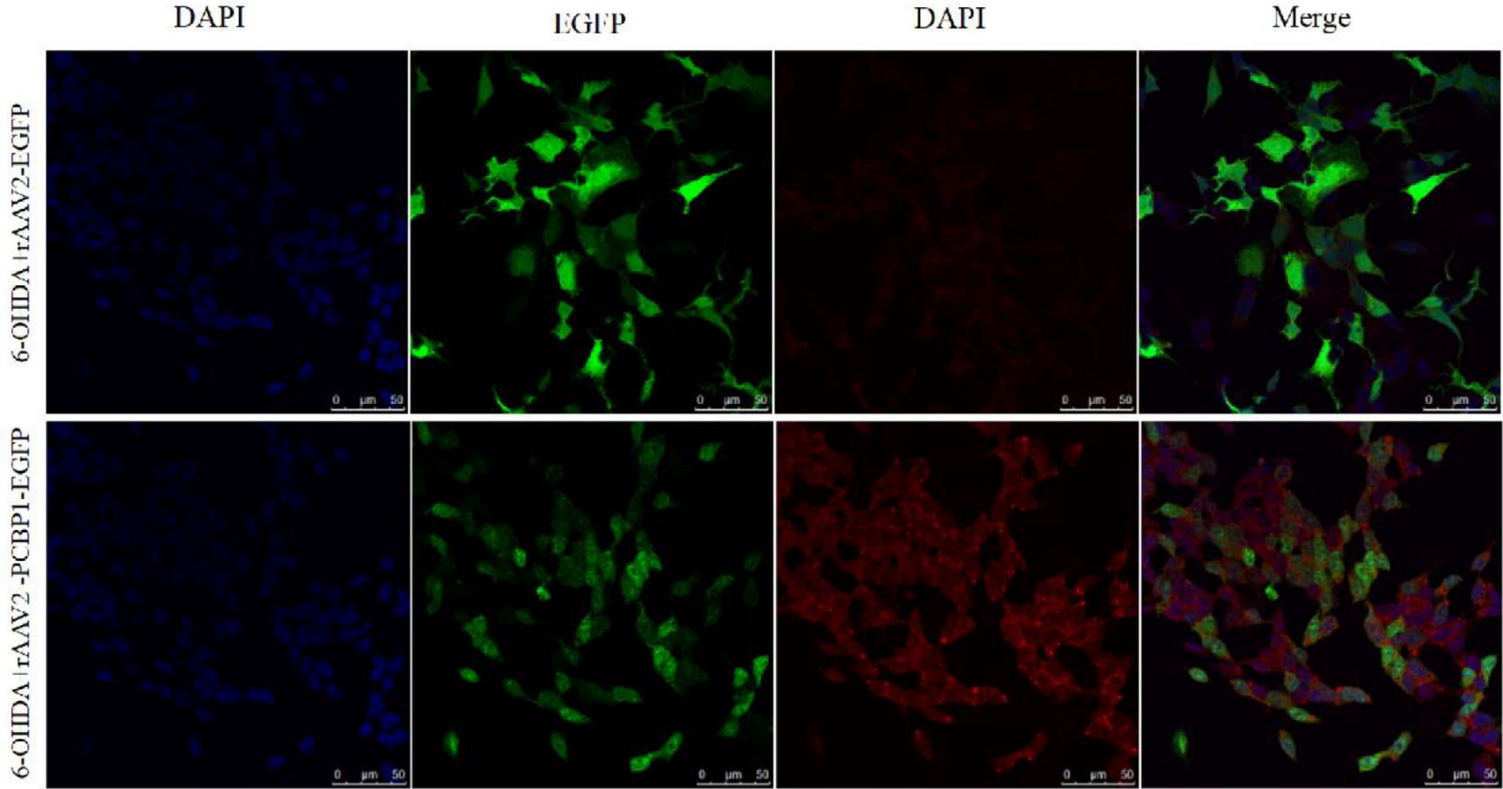
The expression of heat shock protein 70 (HSP70) is enhanced in neuronal cells overexpressing poly(rC)‐binding protein 1 (PCBP1). Fluorescence micrograph of SH‐SY5Y. Left, 4′,6‐diamidino‐2‐phenylindole (DAPI); middle, EGFP; right, merge. Scale bars, 50 μm.

After repeated validation, it has been observed that at the cellular level, overexpression of PCBP1 leads to an increase in intracellular HSP70 expression. Additionally, in the presence of 6‐OHDA, surviving neuronal cells that overexpress PCBP1 exhibit enhanced HSP70 expression (Figure 2).

**FIGURE 3:**
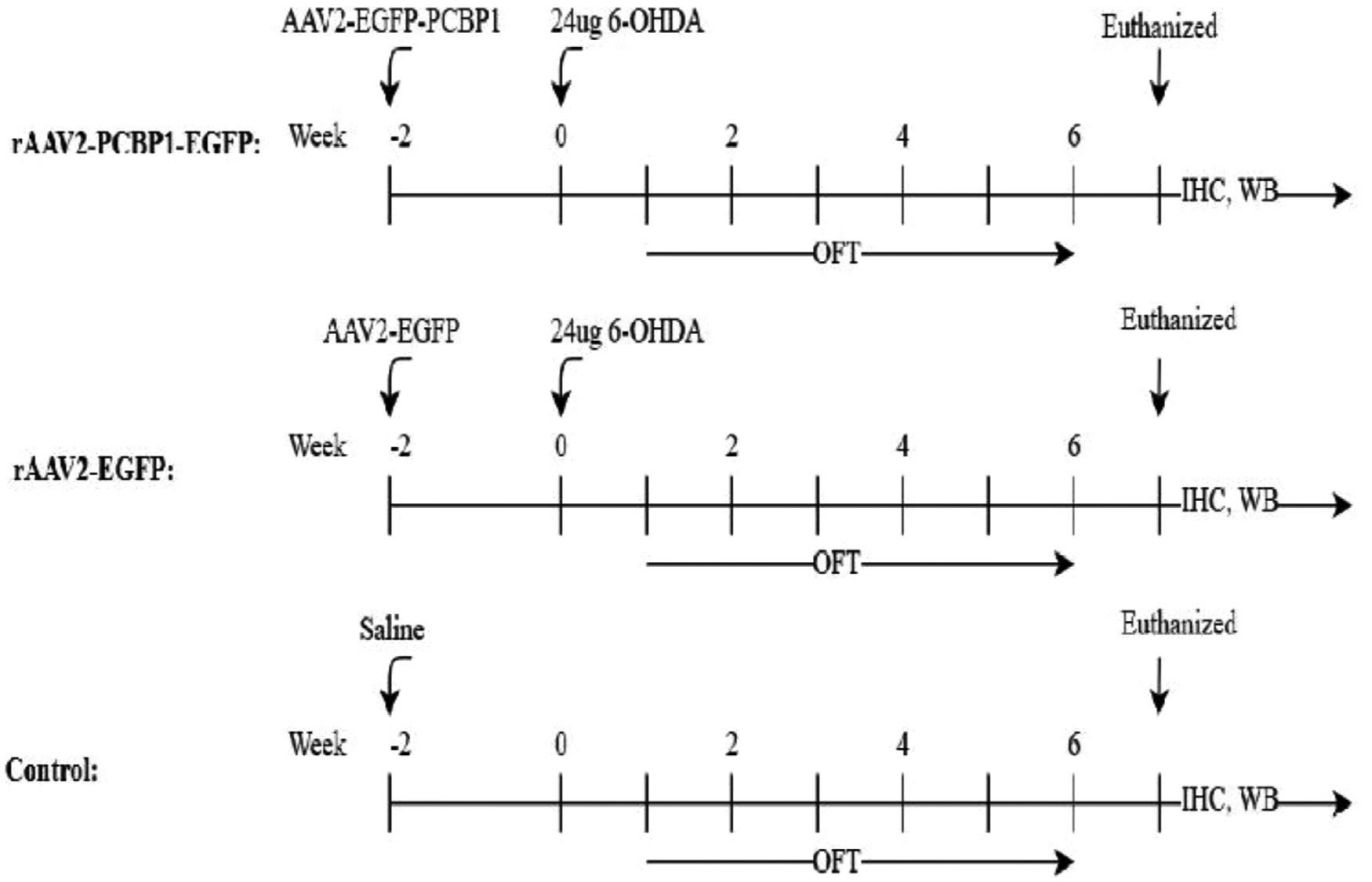
Diagram indicating the time line of the experimental procedures: surgery and testing in three groups of animals.

**FIGURE 4:**
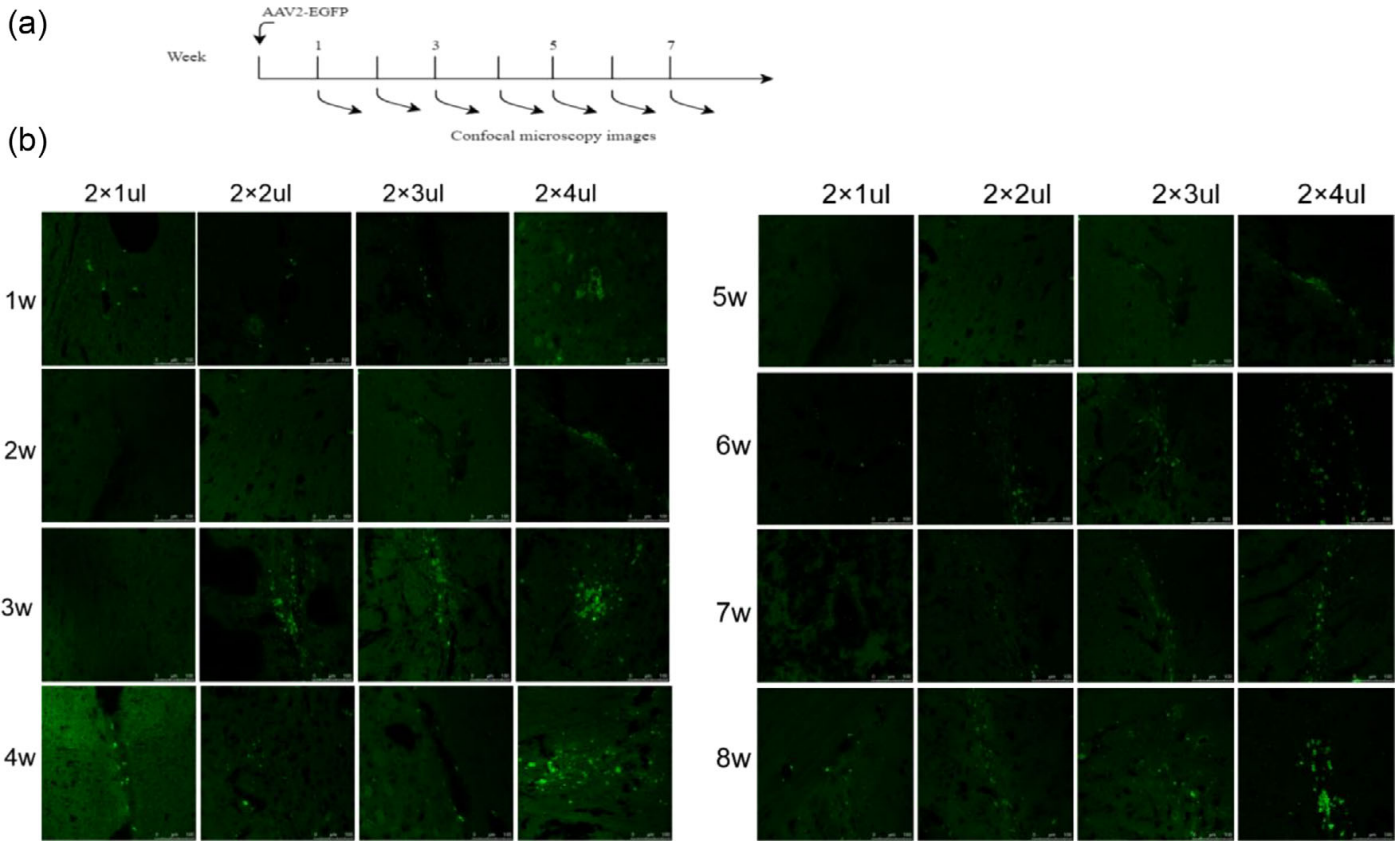
Expression of recombinant adeno‐associated virus (rAAV2)‐EGFP in rats’ brain. (a and b) The titer of rAAV2‐EGFP injected into each group was 2.5 × 108, 5.0 × 108, 7.5 × 108, 1.0 × 109 vg/striatum, one rat in each group was selected at random 1 week after the operation, and the expression of rAAV2‐EGFP in rat brain slices was observed by confocal microscope. *n* = 5–6. Scale bars, 100 μm.

The time line of the experimental procedures for surgery and testing in the three animal groups is also shown in Figure 3. And we further investigated the virus titer‐dependent expression and the time‐dependent expression following gene transfer with rAAV2 vector in Figure 4. The results demonstrated that rAAV2 can maintain stable expression in the striatum and continue for up to 8 weeks after injection. Confocal microscopy images indicated that rAAV2 can achieve stable and persistent expression in the rat brain. Considering the diffusion rate during virus injection, we selected a titer of 5.0 × 10^8^ vg/striatum as the optimal virus dose for neuroprotection. Furthermore, our results demonstrate that rAAV2‐EGFP can be stably expressed in the neurons of the striatum, hippocampus, substantia nigra, and cortex of rats, persisting for up to 8 weeks after injection, providing evidence that rAAV2 can be stably and continuously expressed in rats (Figure S1).

**FIGURE 5:**
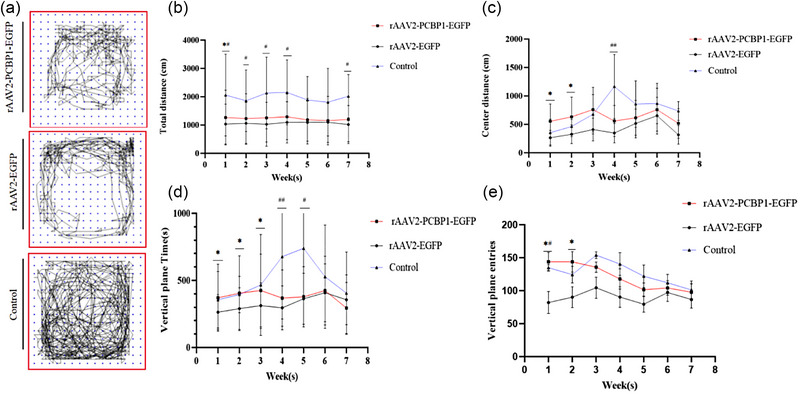
Results of the open field test. (a) Spontaneous movement routes of three groups; (b–e): total distance, center distance, and vertical plane (VP) time/entries. Data are mean  ±  standard error mean (SEM) with *n* =  8–13 per genotype/sex/treatment. Two‐way analysis of variance (ANOVA) with Holm‐Sidak post hoc tests: n.s., not significant; ^*^ *p* < .05, ^**^ *p* < .01 versus control group, ^#^ *p* < .05, ^##^ *p* < .01 versus recombinant adeno‐associated virus (rAAV2)‐EGFP group; data are expressed in terms of mean ± standard error mean (SEM), one‐way ANOVA, and Tukey honestly significant difference (HSD) post hoc test, *n* = 10–15.

The effects of PCBP1 treatment on the non‐motor symptom of 6‐OHDA‐induced anxiety was evaluated using the OFT (Figure 5). The spontaneous movement routes of the three groups are depicted in Figure 4a. The OFT was conducted to analyze motor (total distance and VP time/entries) and neuropsychiatric (center distance) performance of the three groups in Figure 5b–e. We observed a significant decrease in total distance for the rAAV2‐PCBP1‐EGFP and rAAV2‐EGFP rats compared to the control group at 1–4 weeks (*p* < .05), and at 7 weeks (*p* = .032, *F* = 1.62). During the first and second weeks, the rAAV2‐PCBP1‐EGFP group exhibited significantly more entries into the center area and spent more time in the VP (VP time/entries) compared to the rAAV2‐EGFP group (*p* < .05). In the fourth week, the control group showed a significantly higher number of entries into the center area compared to the other two groups (*p* < .01). In the third week, the rAAV2‐PCBP1‐EGFP group had a longer VP time compared to the rAAV2‐EGFP group (*p* < .01), while in the fourth and fifth weeks, the control group had significantly longer VP time compared to the other two groups (*p* < .01, *p* < .01). These results indicate that PCBP1 treatment can influence anxiety‐related behavior in the 6‐OHDA‐induced PD rat model.

**FIGURE 6:**
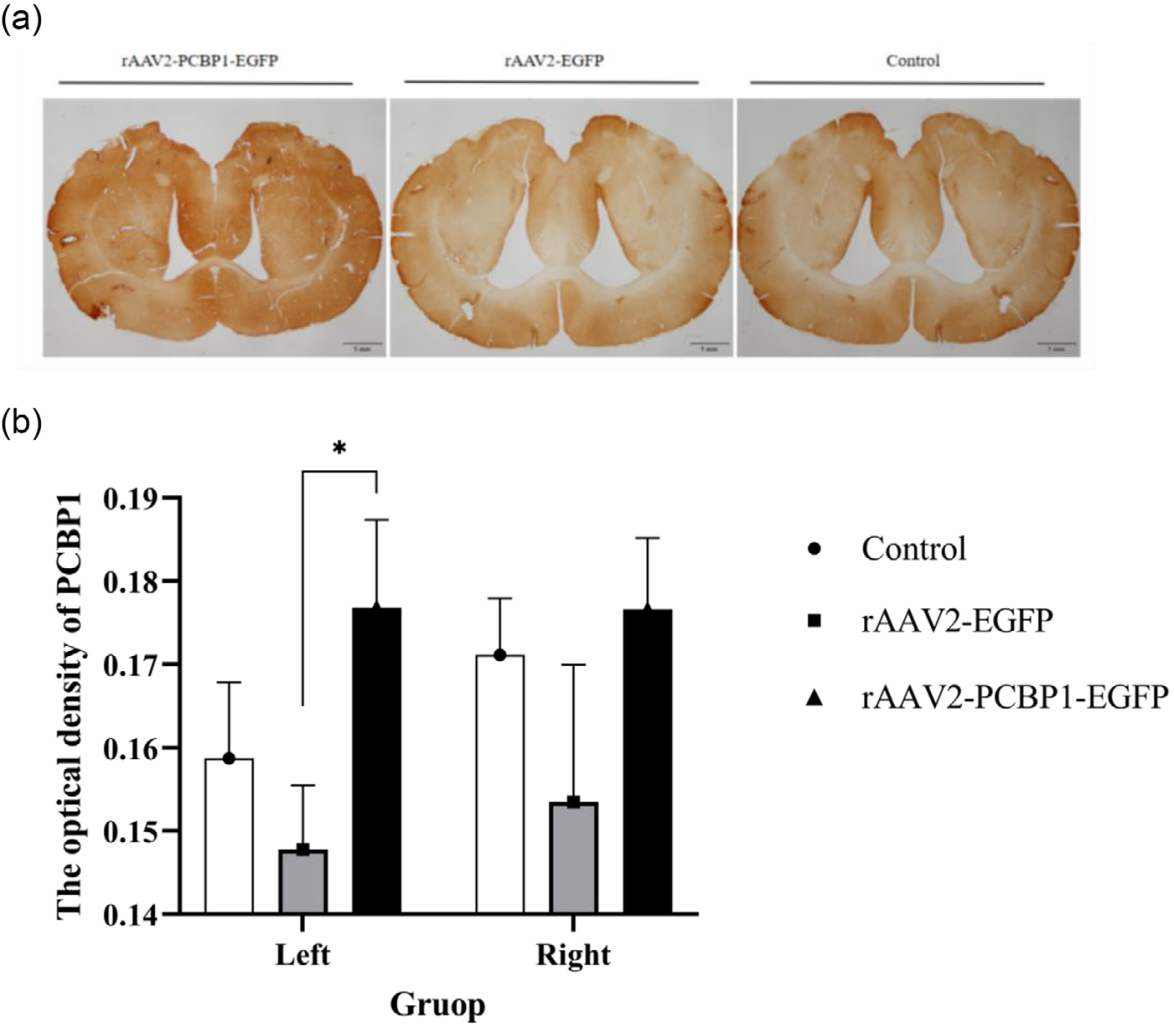
Expression of poly(rC)‐binding protein 1 (PCBP1) in rat brain after recombinant adeno‐associated virus (rAAV2)‐PCBP1‐EGFP injection into the striatum. (a) Expression of PCBP1 in rat striatum of the RAAV2‐PCBP1‐EGFP group, rAAV2‐EGFP group, and control group (immunohistochemical staining); (b) PCBP1 expression in the left and right striatum of each group. ^*^ *p* < .05, one‐way analysis of variance (ANOVA), and Tukey honestly significant difference (HSD) post hoc test, *n* = 10–15. Scale bars, 1 mm.

In Figure 6, we observed that the expression level of PCBP1 in the rAAV2‐PCBP1‐EGFP group showed an increasing trend compared to the other two groups. Additionally, in the left striatum, the level of PCBP1 was significantly higher in the rAAV2‐PCBP1‐EGFP group than in the rAAV2‐EGFP group (*p* < .05).

**FIGURE 7:**
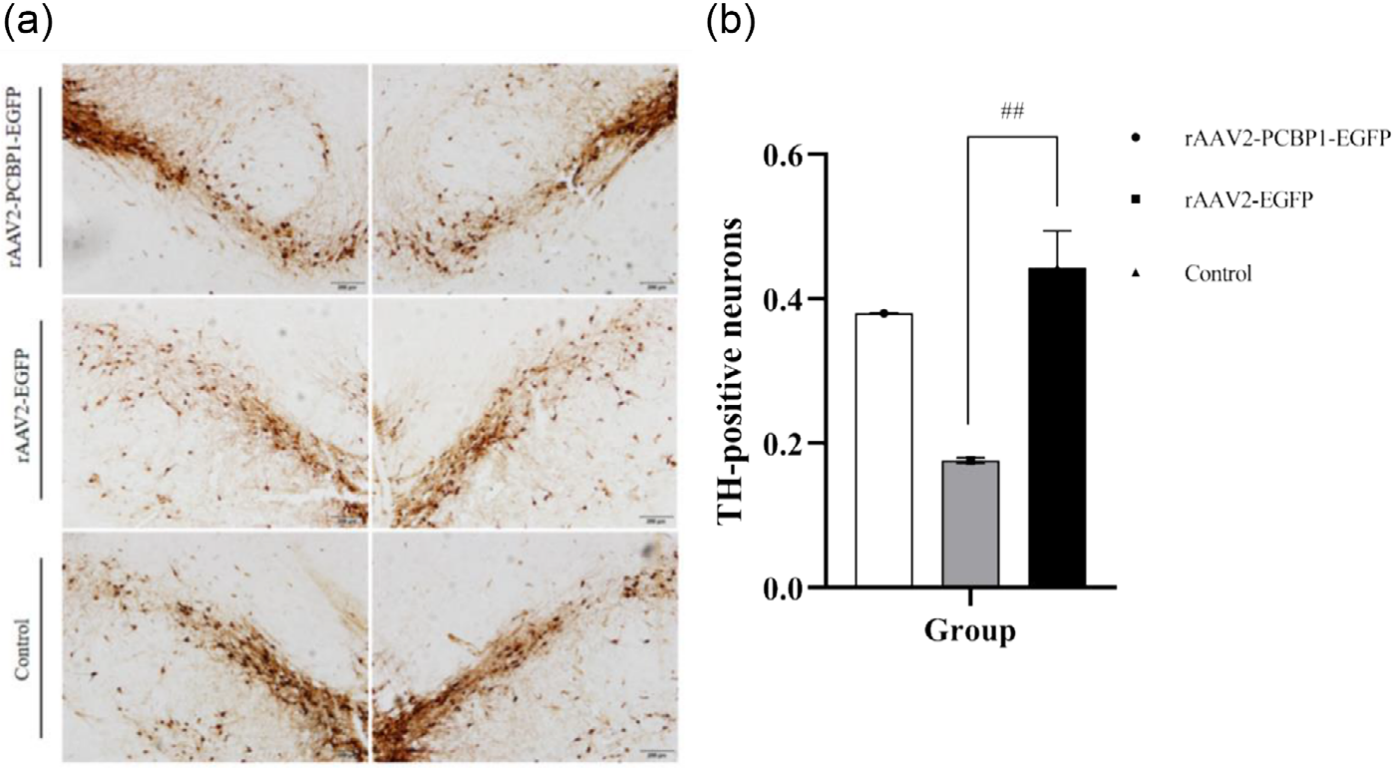
Tyrosine hydroxylase (TH)‐immunoreactivity cells in the SNc. ^##^ *p* < .01 versus recombinant adeno‐associated virus (rAAV2)‐EGFP group. (a) Representative sections showing TH‐reactive cells in the central SNc are visualized; (b) rAAV2‐PCBP1‐EGF was able to protect the nigral TH‐reactive cells in the central SNc, *n* = 10–15. Scale bars, 200 μm.

At 8 weeks post‐lesion, there was an approximately 82% decrease in TH‐reactive neurons in the lesioned SNc in the rAAV2‐EGFP group (Figure 7). When considering all six nigral sections, none of the treatments resulted in significant protection of the TH‐reactive cells. However, rats treated with rAAV2‐PCBP1‐EGFP showed a 53% decrease in cell loss, indicating a trend toward protection of the TH‐immunoreactivity cells (*p* < .01, one‐way ANOVA [*p* = .0023, *F* = 3.275] and Tukey HSD post hoc test).

**FIGURE 8:**
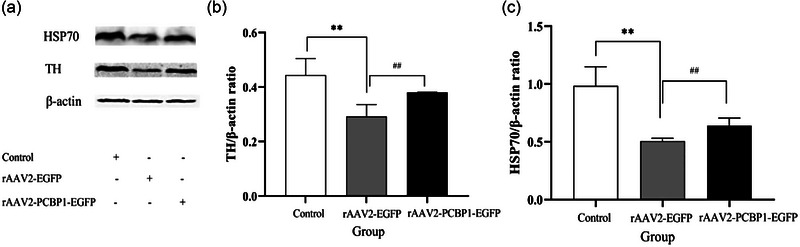
Expression of tyrosine hydroxylase (TH) and heat shock protein 70 (HSP70) in substantia nigra (SN) of each group. Data are expressed in terms of mean ± standard error mean (SEM). ^**^ *p* < .01 versus Control group, ^##^ *p* < .01 versus recombinant adeno‐associated virus (rAAV2)‐EGFP group. *n* = 10–15.

The neuroprotective effects of PCBP1 on neurons in the SNc of 6‐OHDA‐induced rats were explored (Figure 8). In the SNc, there were significant differences in the protein expression levels (*p* < .01) of TH and HSP70 among the three groups. The expression of TH and HSP70 in the control rAAV2‐PCBP1‐EGFP group was significantly lower than that in the control group, with a clear statistical difference (*p* < .01). Additionally, the expression of TH and HSP70 in the rAAV2‐PCBP1‐EGFP group was significantly higher than that in the control rAAV2‐PCBP1‐EGFP group, also with a clear statistical difference (*p* < .01). These results indicate that PCBP1 can promote a protective effect on TH‐immunoreactivity cells in the SNc of 6‐OHDA‐induced PD rats by upregulating the expression of HSP70 in neurons.

## 4 DISCUSSION

In the present investigation, our current research indicates that PCBP1 shows promising neuroprotective effects against PD. Previous experiments have already demonstrated the protective role of PCBP1 in a PD cell model through overexpression. In this study, our objective is to construct a recombinant adeno‐associated vector containing the full‐length ORF of PCBP1 and perform preliminary tests to confirm its protective effect in an in vivo PD animal model. Our ultimate goal is to establish a scientific basis for the potential therapeutic efficacy of PCBP1 in treating PD and to develop corresponding biological molecular agents for PD treatment. These findings contribute to advancing our understanding of PD pathogenesis and offer potential avenues for the development of novel therapeutic strategies targeting PCBP1 to alleviate PD symptoms.

The delivery site is a significant consideration for future gene therapy utilizing neurotrophic factors in PD. When the dopaminergic projections from the substantia nigra to the striatum are compromised, delivering therapeutic agents directly to the substantia nigra becomes challenging. In such cases, alternative transportation routes may be required. In animal models of PD, intrastriatal infusion of a recombinant AAV2‐NRTN vector (CERE‐120) has shown effectiveness in behavioral tests. The neurotrophic factor neurturin (NRTN) immunoreactivity was observed in various brain regions, including the striatum, GP, endopeduncular nucleus, and SNc. These findings highlight the potential of intrastriatal delivery of therapeutic agents as a promising approach for gene therapy in PD and warrant further investigation for clinical application (Kordower et al., 2006).

Many studies have reported neuroprotection in animal models of PD using TH immunocytochemistry to identify dopaminergic cells in the brain, specifically in midbrain centers known to contain concentrations of these cells (Bastide et al., 2015). Given that RBPs are key posttranscriptional regulators, any abnormalities in this family of proteins can modulate the pathophysiology of neurodegenerative diseases by regulating the disease‐associated gene expression (Bastide et al., 2015). For example, TAR DNA binding protein of 43 kDa (TDP‐43) is involved in apoptosis, cell division, and axonal transport through regulation of transcription, alternative splicing, and mRNA stability in frontotemporal dementia, AD, and amyotrophic lateral sclerosis (Gebauer et al., 2021). While Upon PCBP1 treatment, the reduction of TH neurons was found to be less in the disease group. PCBP1 is involved in mRNA binding and stabilization, translational activation or silencing, and iron chaperone function (Mazzio et al., 2004). There are limited reports on the role of PCBP1 in neurodegenerative diseases. For example, PCBP1 has been reported to interact with HSPB1 mutants as well as other genes causing neurological disorders. However, to the best of our knowledge, there has been no report indicating that PCBP1 plays a role in PD. Of particular interest to us is that PCBP1 can upregulate the expression of the HSPA1A gene (Hsp70 protein), which plays a crucial role in combating the misfolding of various functional proteins induced by oxidative stress in PD disease. It has also been reported that overexpression or induction of HSP70 can prevent aTH aggregation and associated toxicity in PD models (Yu et al., 2018). Our research findings on HSP70 in the PD model align with previous studies. It has been reported that the protective effect of HSP70 on substantia nigra striatal degeneration is associated with its anti‐apoptotic activity (Dong et al., 2005). In our prior investigations, we observed a 15.87‐fold upregulation of HSPA6 and a 7.89‐fold upregulation of HSPA1A when PCBP1 was overexpressed in dopaminergic neuronal cell line SH‐SY5Y (Huo & Zhong, 2008). Additionally, we noted that the knockdown of endogenous PCBP1 gene had a significant impact on various genes related to neuronal development, differentiation, and axon formation (Huo et al., 2010).

In our study, we successfully demonstrated that PCBP1 can alleviate the growth restriction induced by 6‐OHDA in SH‐SY5Y cells, and we have also observed that the overexpression of PCBP1 results in an augmentation of HSP70 expression. Subsequently, we investigated the site of 6‐OHDA injection in the rat striatum for administering the rAAV2‐PCBP1‐EGFP virus and assessing its expression in the brain. We established four different titer groups and monitored the expression of rAAV2‐EGFP group every other week for 8 weeks after injection. Our experimental results revealed that the expression of rAAV2‐EGFP started at the first week postinjection and reached its peak at 3–5 weeks, sustaining expression until the eighth week. The rAAV2‐EGFP was found to be expressed in various brain regions, including the striatum, cortex, hippocampus, and substantia nigra, with its expression level being correlated with the administered titer. The expression of PCBP1 in the striatum was relatively lower than that in the hippocampus and cortex, except near the needle passage. This disparity may be attributed to the predominant expression of PCBP1 in the cytoplasm and nuclear membrane of the neuron body. Importantly, we did not observe any adverse immune reactions in any titer group. Based on the previous study, a titer of 5.0 × 10^8^ vg/striatum was chosen as the therapeutic titer for future experiments, aiming to assess its impact on rat behavior and histochemistry. These findings provide valuable insights for further investigations into the potential therapeutic role of PCBP1 in PD.

We observed that the total distance traveled was significantly longer in the control group compared to the lesion group (*p* < .05). Interestingly, rats in the lesion group exhibited different patterns of behavior, characterized by reduced center entries and time spent in the VP, indicative of anxiety‐like behavior. However, rats treated with rAAV2‐PCBP1‐EGFP showed increased center entries in the open field at 1 and 2 weeks, suggesting an improvement in anxiety‐like behavior. However, these differences gradually diminished or disappeared in later weeks, possibly due to the decreasing intensity of drug action over time. Future experiments may require adjustments in the frequency or dosage of drug administration to maintain the intensity of drug action for better evaluation of efficacy.

Furthermore, we compared the expression of PCBP1 and observed increased levels of the target gene expression protein PCBP1 in the PCBP1 treatment group. The content of PCBP1 in the left and right striatum of the same group differed only in the rAAV2‐PCBP1‐EGFP group. Additionally, we observed an increase in TH and HSP70 expression in the PCBP1 treatment group, with differences seen only in the rAAV2‐PCBP1‐EGFP group compared to the rAAV2‐EGFP group and control group. Based on our findings, we cannot help but contemplate that PCBP1 may mitigate protein misfolding (potentially including TH), aggregation induced by toxins, or directly safeguard neurons from damage by upregulating the expression of HSP70. Meanwhile, it is now widely recognized that in the brains of PD models induced by 6‐OHDA, there is a significant increase in the expression of inflammatory cytokines such as TNF‐α and IL‐6. These cytokines have been shown to participate in the process of neurodegeneration by activating NF‐κB (Tiefensee Ribeiro et al., 2021). While Nishinakamura et al. (2007) conducted research revealing that PCBP1 can interact with constitutively activated STAT3 (STAT3), thereby inhibiting the activity of NF‐κB. Furthermore, the overexpression of PCBP1 enhances the antagonistic effect of IL‐10 on IL‐6 (a pro‐inflammatory cytokine), whereas silencing PCBP1 weakens this antagonistic effect. These findings suggest that PCBP1 can participate in the inhibition of the STAT3‐mediated NF‐κB activation pathway. This suggests that inhibiting the activity of NF‐κB could be beneficial for the treatment of PD and other neurodegenerative diseases caused by apoptosis and inflammation. Furthermore, Shi et al. (2008) reported that PCBP1 acts as a molecular chaperone for iron, facilitating the transport of free cytoplasmic iron (both divalent and trivalent) into ferritin for storage and, under certain conditions, releasing bound iron to meet the cytoplasm's iron requirements. As previously mentioned, the increase in free iron within the cytoplasm serves as a trigger for oxidative stress, which can initiate neurodegenerative changes closely linked to the onset of PD. We can hypothesize that even if the total cellular iron content remains unchanged, dysfunctional or misfolded iron‐storing ferritin due to toxin exposure may lead to the excessive release of iron, thereby inducing oxidative stress and causing cellular damage. This is also the direction of our ongoing research: understanding how PCBP1 functions as an iron chaperone and its role in neurological disorders.

Our study confirms the potential of PCBP1 treatment as a therapeutic option for PD, as it shows therapeutic efficacy in both motor‐deficiency and anxiety during PD progression. However, the model used in our study may still require optimization due to complications such as dysphagia and thirst deficiency after the operation, as well as a high mortality rate. Future research should focus on refining the model to improve its validity and reduce potential adverse effects.

Overall, our findings suggest that PCBP1 holds promise as a neuroprotective therapy for PD, addressing both motor and non‐motor symptoms. However, further investigations are warranted to fully understand its mechanisms of action and to optimize the therapeutic approach for PD treatment.

## 5 CONCLUSION

Through our current study, we have successfully demonstrated the neuroprotective role of PCBP1 in the Parkinsonian mouse model. Notably, our research has provided the first evidence confirming the neuroprotective effect of PCBP1 by upregulating the expression of Hsp70 in the PD model induced by 6‐OHDA injection into the striatum. The use of rAAV2‐PCBP1‐EGFP has shown a clear neuroprotective effect on TH in the SNc region of the PD rat model. To further advance this line of research, it will be essential to explore the potential benefits of higher titers, multiple injection sites, different vector serotypes, alternative promoters, and/or varied injection sites for the delivery of PCBP1 treatment. These investigations may enhance the effectiveness and specificity of PCBP1 therapy for PD. Based on our findings, we believe that PCBP1 treatment has significant potential to be developed into a viable therapeutic drug for PD. Its ability to address both motor deficits and anxiety‐like behaviors in PD progression makes it a promising candidate for further development and clinical testing. Continued research in this area could ultimately lead to the development of an effective and comprehensive treatment option for PD.

## AUTHOR CONTRIBUTIONS

**Ling‐Yun Ma**: Writing—original draft; writing—review and editing; data curation. **Lanying Wang**: Formal analysis; writing—original draft. **Jiantao Liang**: Project administration; writing—review and editing. **Lirong Huo**: Writing—review and editing; conceptualization; investigation; funding acquisition.

## CONFLICT OF INTEREST STATEMENT

The authors declare that the research was conducted in the absence of any commercial or financial relationships that could be construed as a potential conflicts of interest.

### PEER REVIEW

The peer review history for this article is available at https://publons.com/publon/10.1002/brb3.3376.

## Supporting information

Supporting Information

Supporting Information

## Data Availability

The data that support the findings of this study are available from the corresponding author upon reasonable request.
