# Supplementary figures and images for: Investigating the neuroprotective potential of rAAV2‐PCBP1‐EGFP gene therapy against a 6‐OHDA‐induced model of Parkinson's disease
Source: Brain Behav. 2024 Jan 29;14(1):e3376. doi: 10.1002/brb3.3376 (PMC10823554; doi:10.1002/brb3.3376)

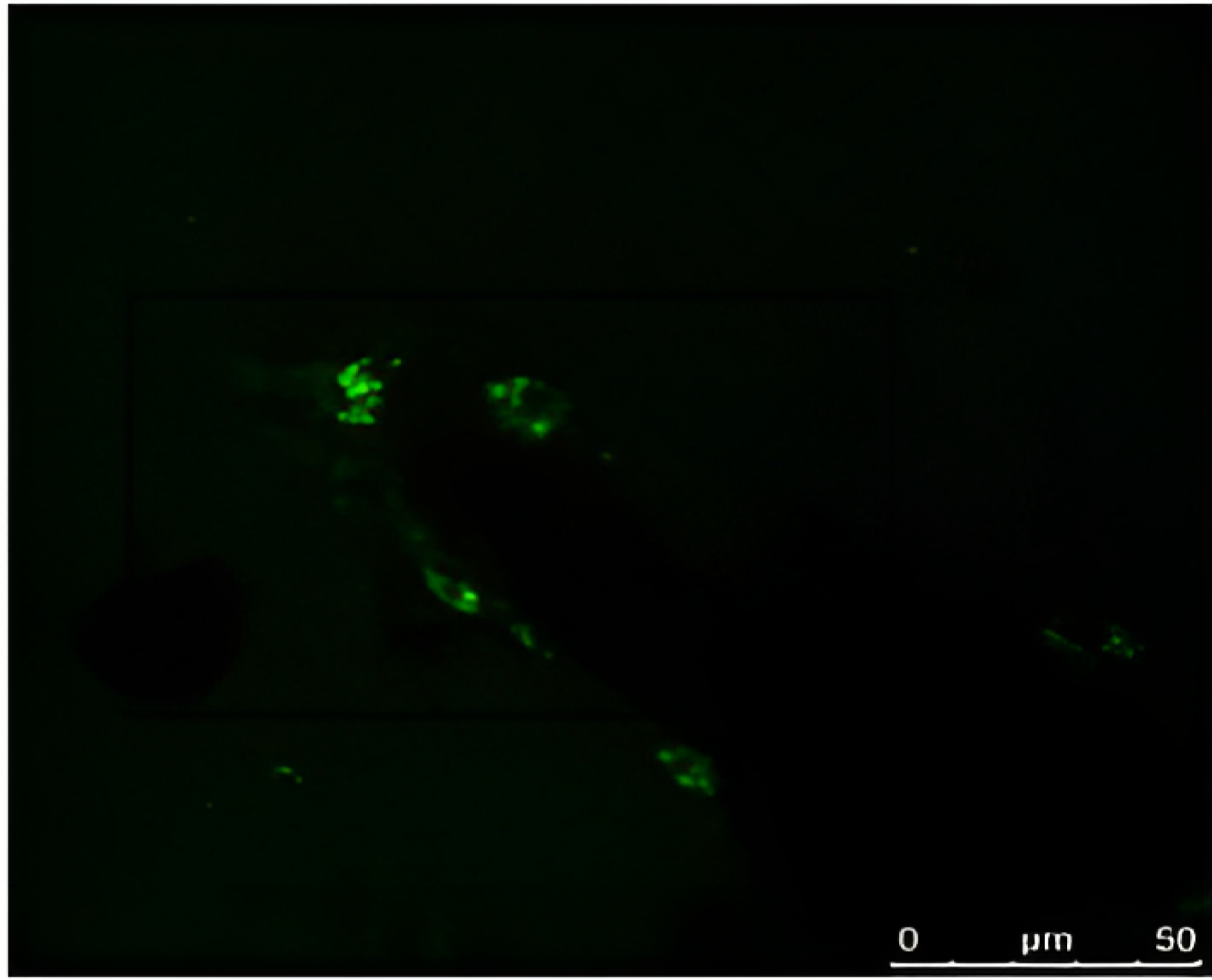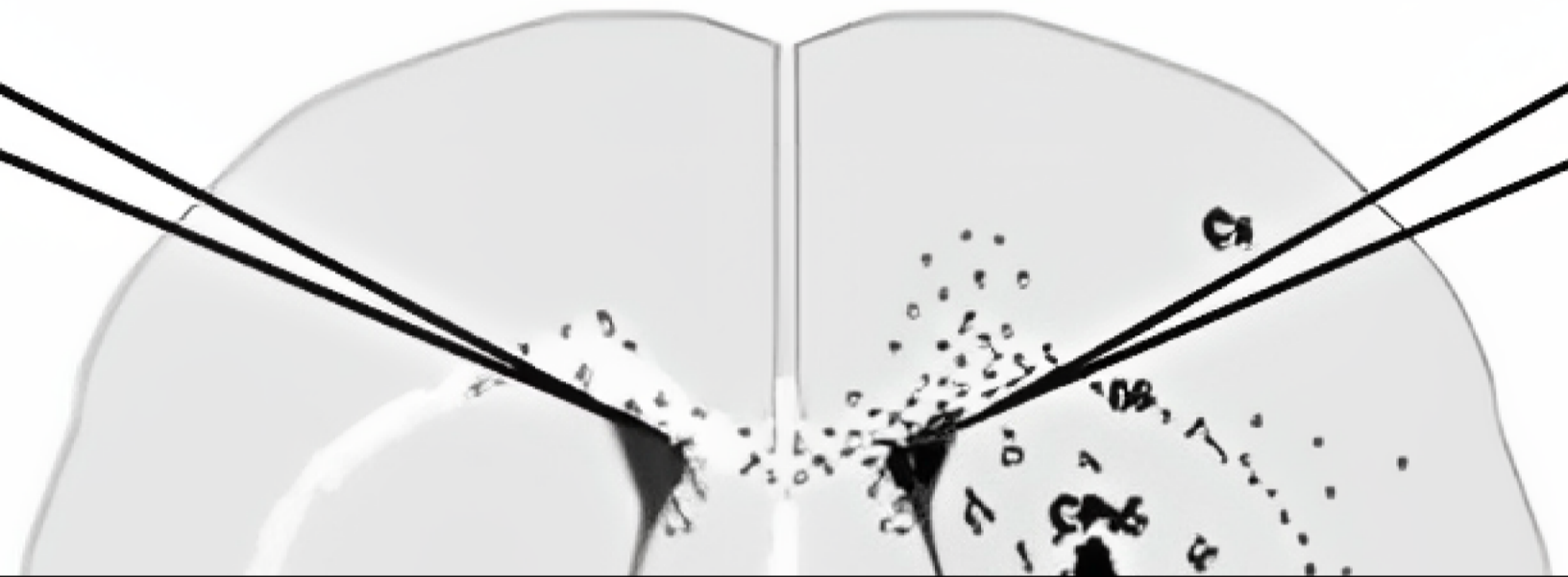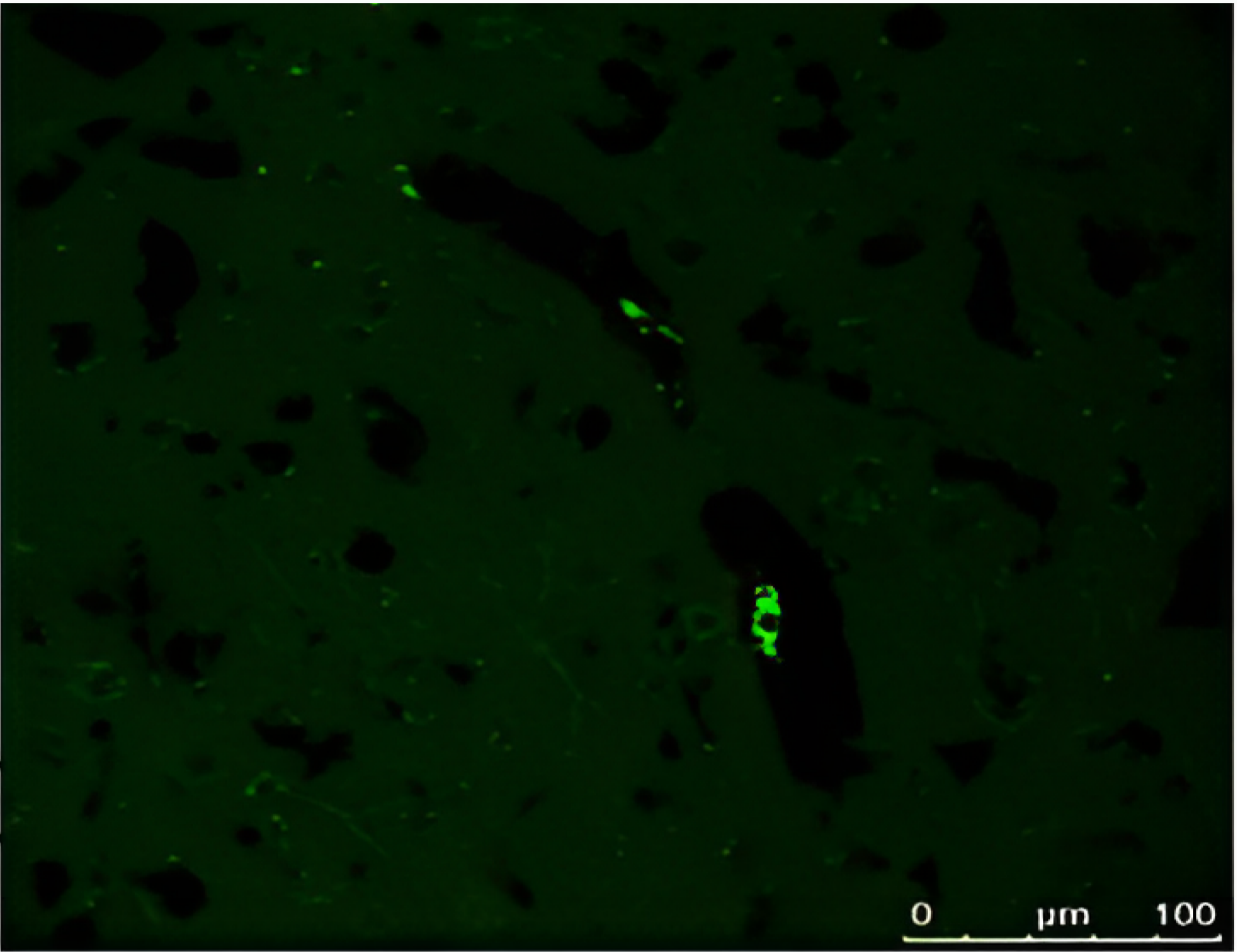

Supplement: Supplementary file 1 — Supporting Information [file BRB3-14-e3376-s001.pdf]
